# A Pharmacokinetic Study of Ephedrine and Pseudoephedrine after Oral Administration of Ojeok-San by Validated LC-MS/MS Method in Human Plasma

**DOI:** 10.3390/molecules26226991

**Published:** 2021-11-19

**Authors:** Sooyoung Lee, Wang-Seob Shim, Heejo Yoo, Sanghee Choi, Jiyoung Yoon, Kwang-Young Lee, Eun-Kyoung Chung, Byung-Cheol Lee, Sung-Vin Yim, Bo-Hyung Kim, Kyung-Tae Lee

**Affiliations:** 1 Department of Biomedical and Pharmaceutical Sciences, Graduate School, Kyung Hee University, Seoul 02447, Korea; ls009@khu.ac.kr (S.L.); tkdgml117@gmail.com (S.C.); yoon02j27y@khu.ac.kr (J.Y.); 2 Kyung Hee Drug Analysis Center, College of Pharmacy, Kyung Hee University, Seoul 02447, Korea; wsshimm@khu.ac.kr (W.-S.S.); heejoyoo93@gmail.com (H.Y.); 3 Department of Pharmaceutical Biochemistry, College of Pharmacy, Kyung Hee University, Seoul 02447, Korea; gloryi@naver.com; 4 Department of Fundamental Pharmaceutical Science, Graduate School, Kyung Hee University, Seoul 02447, Korea; cekchung@khu.ac.kr; 5 Department of Clinical Korean Medicine, Graduate School, Kyung Hee University, Seoul 02447, Korea; hydrolee@khu.ac.kr; 6 Department of Clinical Pharmacology and Therapeutics, Kyung Hee University Medical Center, Seoul 02447, Korea; ysvin@khu.ac.kr; 7 Department of Biomedical Science and Technology, Graduate School, Kyung Hee University, Seoul 02447, Korea; 8 East-West Medical Research Institute, Kyung Hee University, Seoul 02447, Korea

**Keywords:** ephedrine, pseudoephedrine, liquid chromatography-tandem mass spectrometry, bioanalytical method validation, pharmacokinetics, healthy volunteers

## Abstract

A sensitive and reproducible liquid chromatography-tandem mass spectrometry (LC-MS/MS) system was developed and fully validated for the simultaneous determination of ephedrine and pseudoephedrine in human plasma after oral administration of the herbal prescription Ojeok-san (OJS); 2-phenylethylamine was used as the internal standard (IS). Both compounds presented a linear calibration curve (*r*^2^ ≥ 0.99) over a concentration range of 0.2–50 ng/mL. The developed method was fully validated in terms of selectivity, lower limit of quantitation, precision, accuracy, recovery, matrix effect, and stability, according to the regulatory guidelines from the U.S. Food and Drug Administration and the Korea Ministry of Food and Drug Safety. This validated method was successfully applied for the pharmacokinetic assessment of ephedrine and pseudoephedrine in 20 healthy Korean volunteers administered OJS.

## 1. Introduction

As herbal medicines are gaining momentum worldwide [1], the number of patients taking prescribed herbal medicines is simultaneously growing [2]. According to statistical data of the Health Insurance Review and Assessment Service, among 56 herbal prescriptions, Ojeok-san (OJS; wuji-power in China and goshakusan in Japan) was the most commonly prescribed herbal medicine in 2013. OJS is composed of 15 medicinal herbs: *Atractylodes lancea* D.C., *Ephedra sinica* Stapf, *Citrus unshiu* Markovich, *Magnolia officinalis* Rehd. et Wils., *Platycodon grandiflorum* A. DC., *Angelica gigas* Nakai, *Zingiber officinale* Rosc., *Paeonia lactiflora* Pall., *Poria cocos* Wolf, *Cnidium officinale* Makino, *Angelica dahurica* Benth. et Hook. f., *Pinellia ternata* Breit., *Cinnamomum cassia* Presl, *Glycyrrhiza uralensis* Fisch., and *Allium fistulosum* L. In Asian countries, including Korea, China, and Japan, OJS has long been used for treating common colds and illnesses, including gastrointestinal disorders. To date, various studies have reported the analgesic, anti-inflammatory, anti-allergic, and anti-oxidative properties of OJS [3,4,5,6].

It should be noted that, in contrast with synthetic chemical drugs, a single herbal medicine prescription can contain hundreds of active ingredients. Among these ingredients, Ehpedrae Herba, called Ma Huang in China, acts as a diaphoretic to alleviate fever [7]. The chemical components of Ma Huang have been separated and established for approximately 100 years, and the main components include ephedrine alkaloids, such as ephedrine, pseudoephedrine, and methylephedrine. The effect of Ma Huang is mainly attributed to sympathetic stimulation mediated by ephedrine and pseudoephedrine, which increases the activity of α- and β-adrenergic receptors [8]. However, ephedrine interacts with caffeine, amphetamine, nose decongestants, and several other drugs [9,10,11]. In addition, the concentration of ephedrine in rat plasma was found to differ from that following a single ephedrine extract and multi-herbal decoction [12]. Therefore, comprehensively elucidating the pharmacokinetic patterns of ephedrine and pseudoephedrine in human plasma after oral administration of OJS is vital to ensure its safe use.

Methods for estimating ephedrine and pseudoephedrine using high-pressure liquid chromatography (HPLC) with fluorescence detection after derivatization [13] or capillary electrophoresis have been previously reported [14,15]. However, these methods have some limitations, given the complicated sample processing procedure and low sensitivity. In addition, previous studies have described liquid chromatography with tandem mass spectrometry detection (LC-MS/MS), which was reportedly simpler and more sensitive for analyzing six ephedra alkaloids in dietary supplements and biological fluids [16], as well as ephedrine and pseudoephedrine in rat plasma and horse urine [17,18,19]. LC-MS/MS was employed for determining nine herbal phenalkylamines in human plasma, including ephedrine and pseudoephedrine [20]. However, this method was developed with a 10 ng/mL detection limit and was not applied for pharmacokinetic investigations. To our knowledge, no sensitive and reproducible LC-MS/MS method for simultaneous analytical method has been developed for ephedrine and pseudoephedrine in human plasma samples. Furthermore, the development of new and simultaneous ephedrine and pseudoephedrine analytical method was imperative for high-throughput and reliable sample analysis to conduct large-scale pharmacokinetic studies and to routinely measure plasma ephedrine and pseudoephedrine concentrations in the patient. Therefore, the objective of the present study was to develop a fully validated LC-MS/MS method for the simultaneous determination of two main active components, ephedrine and pseudoephedrine, in human plasma. The developed method was then applied to evaluating 20 healthy Korean male volunteers administered OJS powder.

## 2. Results and Discussion

### 2.1. Method Development

#### 2.1.1. Mass Spectrometry

**Figure 1:**
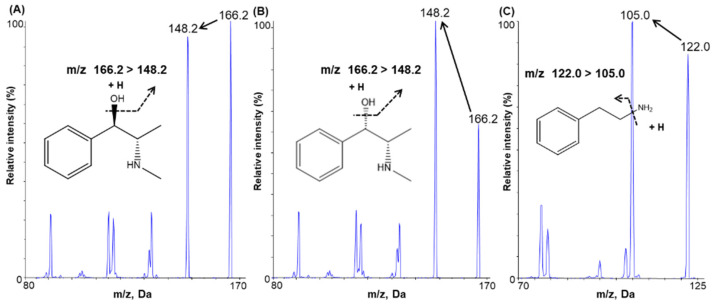
Product ion mass spectra and the pattern of fragmentation of (**A**) ephedrine, (**B**) pseudoephedrine, and (**C**) 2-phenylethylamine (internal standard).

Ephedrine, pseudoephedrine, and IS, at a concentration of 100 ng/mL in methanol, were individually infused into the mass spectrometer using a syringe pump at a continuous flow rate of 10 µL/min to optimize the mass spectrometric conditions and achieve the maximum abundance of product and fragment ions in a positive ionization mode using a Turboionspray ESI interface. Q1 full-scan spectra were characterized by protonated molecules [M + H]^+^ at *m*/*z* 166.2 for ephedrine and pseudoephedrine and *m*/*z* 121.98 for IS. The selected product ions were *m*/*z* 148.2 for both ephedrine and pseudoephedrine; *m*/*z* 105.0 was selected for the IS (Figure 1).

#### 2.1.2. Chromatographic Conditions

Chromatographic conditions, including the column, column temperature, mobile phase composition, and flow rate, were optimized to achieve an adequate peak shape, separation, and run time. In the present study, the following chromatographic columns were evaluated for optimal separation results: Imtakt Cadenza^®^ CD-C18 column (150 × 3.0 mm, 3 µm), Phenomenex Kinetex^®^ C18 column (150 × 4.6 mm, 2.6 µm), DAICEL CHIRALPAK^®^ AGP (150 × 2.0 mm, 5 µm), and Halo^®^ Phenyl-Hexyl column (150 × 2.1 mm, 2.7 µm). Accordingly, the Halo^®^ Phenyl-Hexyl column presented the best results in terms of peak shape, separation, and chromatographic response. The remaining tested columns exhibited poor peak separation. In addition, mobile phases containing different additives, such as formic acid, acetic acid, and ammonium acetate, were evaluated using the gradient method to optimize the peak intensity and chromatographic separation of the two analytes. The most adequate peak shape and selectivity were obtained using 20 mM ammonium acetate adjusted to pH 5 by adding acetic acid to solvent A and methanol for solvent B. Ultimately, the Halo^®^ Phenyl-Hexyl column (150 × 2.1 mm, 2.7 µm) and mobile phase composed of 20 mM ammonium acetate adjusted to pH 5 by adding acetic acid and methanol with gradient elution afforded good separation of the two analytes, exhibiting optimal sensitivity, good resolution, and adequate peak shape, thus satisfying the requirements of sample analysis for pharmacokinetic investigations.

#### 2.1.3. Sample Preparation

According to previous reports [21,22], the liquid-liquid extraction (LLE) method was initially evaluated using MTBE as the extraction solvent. However, given the insufficient recovery, different extraction solvents were evaluated, including methylene chloride, ethyl acetate, *n*-hexane, mixtures of MTBE:methylene chloride (8:2; *v*/*v*), and mixtures of ethyl acetate: *n*-hexane (8:2; *v*/*v*). In addition, sample buffers such as formic acid, hydrochloric acid, and sodium hydroxide, and reconstitution solvents, including methanol and acetonitrile, were assessed to optimize the analyte peak intensity. Ultimately, the highest peak intensity and high analyte recovery were obtained using 3 mL MTBE:methylene chloride (8:2; *v*/*v*) and 20 μL sodium hydroxide (10 mM) as an extraction buffer. Therefore, to meet the requirements for analyzing our 360 plasma samples, we selected one-step LLE using MTBE:methylene chloride (8:2; *v*/*v*) as the extraction solvent.

### 2.2. Method Validation

#### 2.2.1. Specificity and Selectivity

**Figure 2:**
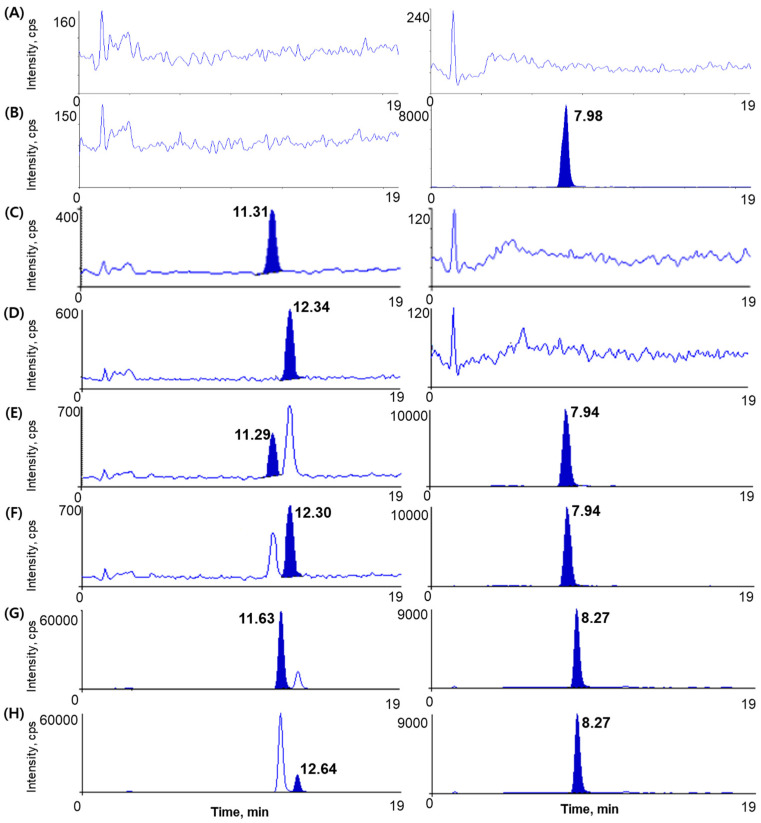
Chromatograms of (**A**) Blank human plasma, (**B**) Blank plasma spiked with IS (5 µg/mL), (**C**) Blank plasma spiked with ephedrine only (0.2 ng/mL, LLOQ), (**D**) Blank plasma spiked with pseudoephedrine only (0.2 ng/mL, LLOQ), (**E**) Blank plasma spiked with ephedrine (0.2 ng/mL) and IS (5 µg/mL), (**F**) Blank plasma spiked with pseudoephedrine (0.2 ng/mL and IS (5 µg/mL), (**G**) Ephedrine (measured concentration 27.23 ng/mL) in sample plasma from a patient 1 h after administering an oral dose of 14.47 g OJS powder and IS (5 µg/mL), and (**H**) Pseudoephedrine (measured concentration 4.08 ng/mL) of sample plasma from a patient at 1 h after administering an oral dose of 14.47 g OJS powder and IS (5 µg/mL).

We employed six different blank plasmas and pooled blank plasmas to verify selectivity at each step. The chromatograms of blank plasma, blank plasma spiked with IS (5 µg/mL), blank plasma spiked with ephedrine (0.2 ng/mL), blank plasma spiked with pseudoephedrine (0.2 ng/mL), blank plasma spiked with ephedrine (0.2 ng/mL), pseudoephedrine, (0.2 ng/mL), and IS (5 µg/mL) showed no interfering peaks at analyte and IS retention times, thus indicating the adequate selectivity of the newly developed analytical method (Figure 2).

#### 2.2.2. Linearity and Lower Limit of Quantification

Using linear regression, calibration curves for ephedrine and pseudoephedrine were plotted with seven concentrations, ranging over 0.2–50 ng/mL. The equation for the calibration curves (*n* = 4) with the mean ± SD of the slope and the intercept was: *y* = 0.251 (±0.014) *x* + 0.007 (±0.009) (*r*^2^ ≥ 0.9906), and *y* = 0.412 (± 0.012) *x* + 0.014 (±0.009) (*r*^2^ *≥* 0.9902) for ephedrine and pseudoephedrine, respectively. The correlation coefficients were greater than 0.99 for all curves, and between-run CVs of response factors were <15% over the assayed concentration range. The signal-to-noise ratio (S/N ratio) of the LLOQ (0.2 ng/mL) suggests a sufficiently sensitive method to quantitate ephedrine and pseudoephedrine in plasma following the oral administration of OJS to human volunteers.

#### 2.2.3. Precision and Accuracy

Table 1 summarizes the intra- and inter-day assay precision and accuracy for ephedrine and pseudoephedrine. The intra-day precision of the method to determine ephedrine and pseudoephedrine concentrations ranged from 5.88% to 14.99% and 8.33% to 10.92%, with an accuracy ranging from 97.10% to 106.40% and 86.78% to 103.70%, respectively. The inter-day precision ranged from 10.97% to 12.53% and from 8.77% to 13.74%, with an accuracy ranging from 91.56% to 94.07% and 89.88% to 93.00%, respectively, for estimating ephedrine and pseudoephedrine concentrations. Thus, all results satisfied the precision and accuracy ranges (%), as specified in the guidance of the MFDS and the FDA for bioanalytical applications [23,24].

#### 2.2.4. Recovery and Matrix Effect

The extraction recovery and matrix effects are summarized in Table 2. Following LLE, the mean extraction recoveries from human plasma at three QC concentrations (0.6, 10, and 40 ng/mL, *n* = 6) were 73.31–76.09% for ephedrine and 71.44–72.97% for pseudoephedrine were. The mean extraction recovery of the IS (5 µg/mL, *n* = 6) was 67.76%. The mean matrix effects for ephedrine at QC concentrations (0.6, 10, and 40 ng/mL, *n* = 6) were 100.48–102.15%, and matrix effects for pseudoephedrine were 101.60–105.17%. These were well within the acceptable limits, suggesting the absence of significant ion enhancement or suppression effects on ephedrine and pseudoephedrine.

#### 2.2.5. Stability

Compared with a freshly prepared stock solution of ephedrine and pseudoephedrine, the mean % peak area of the working solution of ephedrine and pseudoephedrine at room temperature for 7 h was 96.26% and 96.98% and 99.12% and 96.93% at 0.6 and 40 ng/mL, respectively. As shown in Table 3, both ephedrine and pseudoephedrine were stable in plasma for up to 7 h at room temperature, 4 °C, and −70 °C. In addition, the freeze-thaw stabilities of analytes were adequate (85.83–99.50%) after five freeze-thaw cycles. In addition, the samples were stable (87.22–109.60%) in the autosampler (10 °C) for 97 h after LLE. Therefore, based on observed deviations from the nominal concentration within ± 15%, ephedrine and pseudoephedrine were considered stable in human plasma under all examined conditions without substantial degradation.

### 2.3. Application to a Pharmacokinetic Study

The developed and validated analytical method was successfully used to analyze approximately 360 human plasma samples to estimate the pharmacokinetic interaction of OJS in 20 healthy Korean volunteers. The study findings have been previously reported, except for the pharmacokinetics of ephedrine and pseudoephedrine [25]. However, at the time of publication, the concentrations of ephedrine and pseudoephedrine could not be determined simultaneously. Therefore, additional developments were required to overcome analytical difficulties associated with simultaneous quantification.

**Figure 3:**
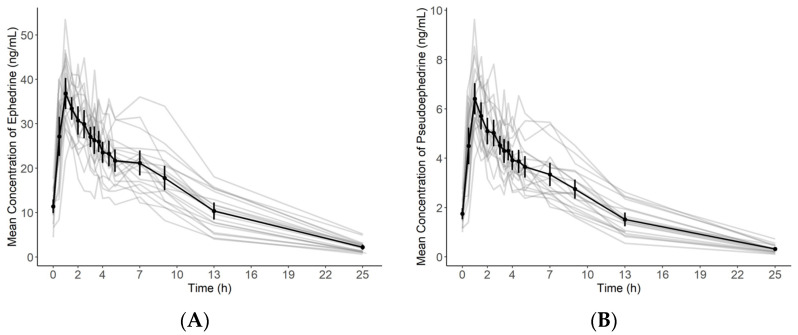
Mean (±standard deviation) plasma concentration-time profile of ephedrine and pseudoephedrine in the plasma samples of 20 healthy volunteers after the oral administration of OJS powder overlaid on gray individual spaghetti plots: (**A**) Ephedrine; (**B**) Pseudoephedrine.

Figure 3 shows the mean ± SD (or individual) plasma concentration-time curve of ephedrine and pseudoephedrine in the plasma samples of 20 healthy volunteers after oral administration of OJS powder. The pharmacokinetic parameters of ephedrine and pseudoephedrine are listed in Table 4. We thought that steady-state was already reached on day 5 after the first OJS dose, given the five half-lives of each compound. Therefore, for calculating pharmacokinetic parameters, the concentrations of blood samples on days 5 and 7 were used as 0 h pre-OJS dosing and 0.5 h post-OJS dosing concentrations, respectively.

The concentrations of ephedrine from 8 subjects and pseudoephedrine from all subjects were below the LLOQ at 49 h after the last OJS dose. In contrast, all concentrations of both components were detectable at 25 h. Thus, 25 h was used as the last concentration time to calculate the AUC_last_. Additionally, the mean ± SD of AUC_last_ of ephedrine, including 49 h, was 353.78 ± 101.42; this value did not considerably differ from the AUC_last_ of ephedrine, excluding 49 h (336.88 ng·h/mL).

Previously, the mean ± SD of half-life of ephedrine were 5.7 ± 2.2 h in a pharmacokinetics study assessing 12 healthy volunteers who administered 20 mg ephedrine tablet [26]. In another pharmacokinetics study for 16 volunteers who administered 120 mg pseudoephedrine capsule, the mean ± SD of half-life of pseudoephedrine were 5.9 ± 2.2 h [27]. The studies of traditional medicine containing ephedrine and pseudoephedrine were also similar to those reported in this study. In a study assessing six healthy volunteers who administered 0.6 g Ma Huang preparation, the mean ± SD of half-lives of ephedrine and pseudoephedrine were 5.6 ± 1.2 and 4.9 ± 0.9 h, respectively [28]. Another study examining 12 healthy volunteers who administered Ma Huang tang 350 mL (contained Ma Huang 18 g) has reported mean ± SD of half-lives of 4.2 ± 1.0 h and 4.3 ± 0.8 h for ephedrine and pseudoephedrine, respectively [29]. No report has addressed the pharmacokinetics of ephedrine and pseudoephedrine in OJS-dosed humans, especially on reaching steady-state. Therefore, this study may be valuable for clarifying the pharmacokinetic characteristics of ephedrine and pseudoephedrine in OJS. In addition, our study found that the half-lives of ephedrine and pseudoephedrine in the current study were similar to those reported in previous studies regardless of drug formulation.

In addition, further studies are needed to analyze ephedrine and pseudoephedrine together with their metabolites, norephedrine and norpseudoephedrine, respectively, in human plasma after OJS administration, although these metabolites are only small amounts [30,31,32,33]. These data will give valuable information on the comparative ephedrine and pseudoephedrine metabolism after pure or herbal formulation administration. In this study, there is a limitation for completely determining metabolite pharmacokinetics after OJS administration. Therefore, further analysis and pharmacokinetic studies, including norephedrine and norpseudoephedrine analysis, can help to better understand the metabolic properties of OJS and to use it safely.

## 3. Materials and Methods

### 3.1. Chemicals and Reagents

Ephedrine (purity 99.7%) and pseudoephedrine (purity 99.9%) were obtained from the Ministry of Food and Drug Safety in Korea (MFDS). 2-phenylethylamine (internal standard [IS], purity 100%), methyl tert-butyl ether (MTBE), and methylene chloride were purchased from Sigma-Aldrich (St. Louis, MO, USA). High-performance liquid chromatography (HPLC)grade acetonitrile and methanol were purchased from J.T. Baker (Phillipsburg, NJ, USA). A Milli-Q^®^ water purification system (Millipore Co., MA, USA) was used to obtain purified water for HPLC analysis. All other chemicals and solvents used were of the highest available analytical grade. OJS was provided by Hanpoong Pharmaceutical and Food Co. Ltd. (Wanju, Korea). The content of ephedrine, pseudoephedrine, hesperidin, paeoniflorin, cinnamic acid, and glycyrrhizic acid was 1.2, 0.16, 5.7, 3.4, 64.8, and 1.2 mg/g crude material, respectively.

### 3.2. Instrumentation and Chromatographic Conditions

Liquid chromatography was performed on a Shimadzu Nexera X2 (Shimadzu, Kyoto, Japan), and chromatographic separation was carried out using a Halo^®^ Phenyl-Hexyl column (150 × 2.1 mm, 2.7 µm; Advanced Materials Technology, Wilmington, DE, USA). The mobile phase consisted of 20 mM ammonium acetate (pH 5.0, adjusted with acetic acid) and 100% methanol with a gradient method. The gradient program was as follow: 3% B at 0–2 min, 3–13% B at 2–14 min, 13% B at 14–17 min, 13–3% B at 17–17.5 min, 3% B at 17.5–19 min. The flow rate was set at 250 µL/min. Mass spectrometry detection was performed on an API 4000 triple-quadrupole mass spectrometer (Applied Biosystems SCIEX, Framingham, MA, USA) equipped with an electrospray ion source. Figure 1 shows the MS/MS spectra of ephedrine, pseudoephedrine, and IS with their fragmentation patterns. The optimized source parameters of ephedrine, pseudoephedrine, and IS are listed in Table 5. The analytical data were processed using Analyst^®^ 1.6.2. software (AB SCIEX, Concord, ON, Canada).

### 3.3. Preparation of Calibration Standards and Quality Control Samples

Primary stock solutions of ephedrine and pseudoephedrine were prepared at a concentration of 1 mg/mL in dimethyl sulfoxide (DMSO) and IS was dissolved in deionized water at the same concentration. They were further diluted with 50% methanol (*v*/*v*) to obtain working solutions at several concentrations and stored at −20 °C. Calibration samples were prepared by spiking ephedrine and pseudoephedrine in blank plasma to obtain the following concentrations: 0.2, 0.5, 2, 5, 10, 25, and 50 ng/mL. Quality control (QC) samples were prepared in the same manner as the calibration standards to achieve low, medium, and high concentrations of 0.6, 10, and 40 ng/mL. Calibration and QC samples were freshly prepared on each day of analysis.

### 3.4. Plasma Sample Preparation

Plasma samples were stored in a freezer at −70 °C and thawed at room temperature before processing. An aliquot of each plasma sample (200 μL) was placed in a borosilicate glass disposable culture tube. IS (20 μL, 5 μg/mL of 2-phenylethylamine), 10 mM sodium hydroxide (20 μL), and MTBE:methylene chloride = 8:2 (*v*/*v*) (3 mL) were added, followed by vortexing for 10 min. After centrifugation at 3081× *g* for 10 min, 2.7 mL of the supernatant was transferred to a clean glass culture tube and evaporated to dryness under N_2_ gas at 50 °C. The residues were reconstituted with 200 μL of 50% methanol (*v*/*v*), and 15 μL was injected into the analytical column for analysis.

### 3.5. Method Validation

The developed method was validated in compliance with bioanalytical method validation guidelines published by the Ministry of Food and Drug Safety in Korea (MFDS) and the U.S. Food and Drug Administration (FDA) [23,24].

#### 3.5.1. Specificity and Selectivity

To assess the selectivity of the method, six randomly selected blank human plasma samples of different origins were analyzed for the potential interference of endogenous compounds simultaneously eluted at the retention times of the analyte and the IS. No interfering peaks were observed, suggesting acceptable selectivity of the developed method.

#### 3.5.2. Linearity and Lower Limits of Quantification

The calibration curve of standards was established using seven ephedrine and pseudoephedrine concentrations (0.2, 0.5, 2, 5, 10, 25, and 50 ng/mL). Linearity was evaluated by plotting the peak area ratios (*x*) of the standard to IS versus the concentrations of the standard (*y*) using weighted (1/*x*^2^) linear least-squares regression (*y* = *ax* + *b*) of the plasma concentrations and the measured peak area ratios. A calibration curve with a correlation coefficient (*r*^2^) of 0.99 or greater was deemed to have adequate linearity. The lower limit of quantification (LLOQ) was defined as the lowest concentration on the calibration curve with a signal-to-noise ratio (S/N) greater than 10. The acceptance precision and accuracy criteria for each back-calculated standard concentration were ±15% deviation from the nominal value, except at LLOQ and ±20% at LLOQ.

#### 3.5.3. Precision and Accuracy

Inter- and intra-day precision and accuracy were evaluated by analyzing five replicates at four different concentrations (0.2, 0.6, 10, and 50 ng/mL) on three consecutive days (one run per day). The mean and standard deviation (SD) were estimated for the calculated concentrations over these batches. Accuracy and precision were presented as relative error (RE) and coefficient of variation (CV), respectively. The accuracy and precision were considered sufficient if RE and CV were within ±15% for each nominal concentration except at LLOQ, where RE and CV should be within ±20%.

#### 3.5.4. Extraction Recovery and Matrix Effect

The extraction recovery and matrix effect were evaluated by assessing the ion suppression or enhancement caused by the plasma matrix during analysis. They were analyzed based on the analytes in the pre-extraction spiked matrix ([A]), analytes in the post-extraction spiked matrix ([B]), and pure analyte solutions in 50% methanol (*v*/*v*) ([C]). The recovery of ephedrine, pseudoephedrine, and the IS at three QC concentrations was evaluated by comparing the peak areas of [A] to the peak areas of [B], which represented 100% recovery. The matrix effect of ephedrine, pseudoephedrine, and the IS was assessed by comparing the peak areas of [B] with the peak areas of [C], and the percent ratio (B/C ×100%) was used to estimate the matrix effect.

#### 3.5.5. Stability

The stability of the working solution was tested using three replicates of low-and high-concentration QC samples by comparing their peak areas with those of freshly prepared stock solutions. The working solutions were stored at room temperature for 7 h. The stability of ephedrine and pseudoephedrine in plasma was examined under the following different conditions using replicates (*n* = 3, at each concentration) of QC (0.6, 10, and 40 ng/mL): short-term stability at room temperature, 4 °C, and −70 °C for 7 h, freeze-thaw stability after five freeze-thaw cycles at −70 °C, and autosampler stability at 10 °C for 97 h.

### 3.6. Application to a Pharmacokinetic Study

The analytical method described above was applied to analyze plasma samples obtained from a pharmacokinetic interaction study in healthy volunteers. An open-label, 1-sequence, 2-period, 2-treatment sequential crossover study was conducted in accordance with the Declaration of Helsinki and Korean Good Clinical Practice (Clinical Research Information Service, CRIS; https://cris.nih.go.kr (accessed on 3 November 2021) Registry Number: KCT0002447). The study was performed at the Clinical Trial Center, Kyung Hee University Hospital, Seoul, Korea. Among the 22 healthy male volunteers enrolled in this study, 20 completed the study.

OJS powder 14.47 g/pack was administered three times per day from the first day of OJS administration to the 7th day. On the 8th day, the last OJS was administered once. Blood samples were collected before OJS administration on the 5th day and after 0.5 h of OJS administration on the 6th and 7th days. On the 8th day, blood samples were collected at 1, 1.5, 2, 2.5, 3, 3.33, 3.67, 4, 4.5, 5, 7, 9, 13, 25, and 49 h after the administration of the last OJS dose. The blood samples were centrifuged immediately for 10 min, and the plasma was harvested and stored at −70 °C until required for LC-MS/MS analysis.

Pharmacokinetic parameters were calculated using R version 4.1.0 (Vienna, Austria) [34] with the PKNCA version 0.9.4 library [35]. The peak plasma concentration (C_max,ss_) at steady-state and the time to reach the C_max,ss_ (T_max_) of ephedrine and pseudoephedrine were determined using individual plasma drug concentration-time profiles on the 8th day after the first OJS administration. The area under the plasma drug concentration-time curve from the last OJS administration to the last value above the limit of quantification (AUC_last_) and from the last OJS dose to 8 h during the dosing interval at steady-state (AUC_τ,ss_) of ephedrine and pseudoephedrine were calculated using the linear-up log-down trapezoidal method. The terminal elimination rate constant (k_z_) was estimated from the regression of the log-linear decrease in the plasma concentration-time profile, and the terminal elimination half-life (t_1/2_) was calculated from the natural logarithm of 2 divided by k_z_.

## 4. Conclusions

The newly developed LC-MS/MS method, which is simple and sufficiently sensitive, was comprehensively validated according to the MFDS and the U.S. FDA guidelines and was successfully used to simultaneously determine ephedrine and pseudoephedrine in human plasma following the oral administration of OJS powder (14.47 g/pack) with water. Given differences observed in plasma ephedrine concentrations on administering a single ephedrine extract and a multi-herbal preparation, our findings may be valuable for clarifying the pharmacokinetic characteristics of ephedrine and pseudoephedrine in OJS.

## Figures and Tables

**Table 1:** Intra- and inter-day precision and accuracy of ephedrine and pseudoephedrine (*n* = 5).

Compound	Nominal Concentration (ng/mL)	Intra-Day (*n* = 5)					Inter-Day (*n* = 5)				
		Mean ± SD (ng/mL)			Precision (CV, %) ^a^	Accuracy (%) ^b^	Mean ± SD (ng/mL)			Precision (CV, %)	Accuracy (%)
Ephedrine	0.2	0.19	±	0.03	14.99	97.10	0.19	±	0.02	11.38	94.07
	0.6	0.60	±	0.05	8.85	100.57	0.55	±	0.06	11.09	91.56
	10	10.26	±	0.63	6.09	102.62	9.21	±	1.01	10.97	92.09
	50	53.20	±	3.13	5.88	106.40	46.20	±	5.79	12.53	92.40
Pseudo- ephedrine	0.2	0.21	±	0.02	9.32	103.70	0.19	±	0.03	13.74	93.00
	0.6	0.55	±	0.06	10.92	90.80	0.54	±	0.05	8.77	89.88
	10	8.68	±	0.72	8.33	86.78	9.15	±	0.83	9.02	91.53
	50	43.47	±	3.68	8.45	86.95	45.39	±	4.47	9.84	90.78

^a^ CV (%) = (standard deviation of calculated concentrations/mean concentration) × 100. ^b^ Accuracy (%) = (predicted con-centration/nominal concentration) × 100.

**Table 2:** Extraction recovery and matrix effect of ephedrine, pseudoephedrine, and IS.

Compound	Nominal Concentration (ng/mL)	Recovery (%) ^a^				Matrix Effect (%) ^b^			
		Mean ± SD (%)			CV (%)	Mean ± SD (%)			CV (%)
Ephedrine	0.6	76.09	±	4.97	6.53	100.48	±	3.84	3.82
	10	76.04	±	2.17	2.86	101.99	±	2.87	2.82
	40	73.31	±	4.14	5.65	102.15	±	2.03	1.98
Pseudo- ephedrine	0.6	72.76	±	4.14	5.69	105.17	±	2.98	2.83
	10	72.97	±	2.43	3.34	101.60	±	1.33	1.31
	40	71.44	±	3.19	4.47	102.56	±	1.83	1.79
IS	5000	67.76	±	10.13	14.95	103.70	±	10.91	10.52

^a^ Extraction recovery (%) = [(peak area of analyte spiked before extraction)/(peak area of analyte spiked after extraction)] × 100. ^b^ Matrix effect (%) = [(peak area of analyte spiked after extraction)/(peak area of analyte in the pure standard solution)] × 100. Data are presented as mean ± SD (*n* = 6).

**Table 3:** Stability data for ephedrine and pseudoephedrine in human plasma samples (*n* = 3).

Nominal Concentration (ng/mL)	Working Solutions (Mean ± SD, %)			Plasma Samples (Mean ± SD, %)														
	Room Temperature (7 h)			Room Temperature (7 h)			4 °C (7 h)			−70 °C (7 h)			Freeze-Thaw Stability (5 Cycles)			Autosampler (97 h, 10 °C)		
**Ephedrine**																		
0.6	96.26	±	2.57	104.83	±	6.95	100.61	±	1.95	109.00	±	10.58	92.28	±	4.73	103.89	±	10.00
10				113.09	±	4.03	108.05	±	4.45	112.71	±	11.06	99.50	±	3.67	87.22	±	9.49
40	96.98	±	1.61	102.04	±	6.27	102.64	±	4.78	103.25	±	2.67	93.46	±	3.32	109.60	±	14.05
**Pseudoephedrine**																		
0.6	99.12	±	1.42	98.39	±	2.28	93.06	±	7.42	96.83	±	7.26	85.83	±	7.78	104.11	±	10.73
10				108.49	±	6.78	103.17	±	1.77	109.58	±	10.87	95.13	±	5.60	88.64	±	7.12
40	96.93	±	2.24	91.35	±	5.95	91.73	±	4.66	92.92	±	4.33	91.73	±	2.40	106.25	±	16.45

**Table 4:** Pharmacokinetic parameter estimates of ephedrine and pseudoephedrine after the oral administration of OJS powder. (*n* = 20).

Parameter	Ephedrine (Mean ± SD)			Pseudoephedrine (Mean ± SD)		
C_max__,ss_ (ng/mL)	39.24	±	6.45	6.83	±	1.19
AUC_last_ (ng·h/mL)	336.88	±	86.13	52.92	±	13.48
AUC_τ,ss_ (ng·h/mL)	180.49	±	31.59	30.08	±	5.33
T_max_ (h)	1.36	±	1.21	1.44	±	0.85
t_1/2_ (h)	5.98	±	1.23	5.20	±	0.65

C_max,ss_, peak plasma concentration at steady-state; AUC_last_, area under the plasma drug concentration-time curve to the last measurable time; AUC_τ,ss_, area under the plasma drug concentration-time curve during dosing interval at steady-state; T_max_, time to reach the peak plasma concentration; t_1/2_, terminal half-life.

**Table 5:** Optimized MRM parameters and retention time for the determination of ephedrine, pseudoephedrine, and the IS.

Compound	Ion Transition (*m*/*z*)	DP (V)	EP (V)	CE (V)	CXP (V)	RT (Min)
Ephedrine	166.20 → 148.20	40.0	6.0	15.0	8.0	11.5
Pseudoephedrine	166.20 → 148.20	40.0	6.0	15.0	8.0	12.5
IS (2-phenylethylamine)	121.98 → 105.00	111.0	10.0	17.0	18.0	8.3

DP, declustering potential; EP, entrance potential; CE, collision energy; CXP, cell exit potential; RT, retention time.

## Sample Availability

Samples of the compounds are not available from authors.
